# Impact of Ghana’s fee exemption policy on maternal health service utilisation: an inverse probability of treatment weighting analysis of pooled national data

**DOI:** 10.7189/jogh.15.04058

**Published:** 2025-02-21

**Authors:** Kennedy Mensah Osei, Andreana Ayiilaboro Awog-badek, Danik Iga Prasiska, Durga Datta Chapagain, Min Jin Ha

**Affiliations:** 1 Department of Global Health Security, Graduate School of Public Health, Yonsei University, Seoul, South Korea; 2 International Health and Tropical Medicine, Nuffield Department of Medicine, University of Oxford, Oxford, UK; 3 Department of Health Informatics and Biostatistics, Graduate School of Public Health, Yonsei University, Seoul, South Korea

## Abstract

**Background:**

Fee exemption policies are key strategies for reducing the barriers to accessing maternal health services and improving maternal and child health outcomes. This study used pooled national data to determine the impact of Ghana’s user fee exemption policy on maternal health service utilisation since it was implemented in 2008.

**Methods:**

Using four rounds of cross-sectional data from national surveys on women with live births, we conducted an inverse probability of treatment weighting analysis to evaluate the causal effects of Ghana’s user fee exemption policy intervention on the timing of first antenatal care (ANC) visit, completion of four or more ANC visits and facility-based delivery as indicators of maternal health service utilisation.

**Results:**

The average treatment effect of the fee exemption policy was an increase of 8, 9, and 21% in the utilisation of timely first ANC visit, completion of the recommended number of ANC visits, and facility-based delivery, respectively. Wealth index categorisation showed a clear stepwise increase in the likelihood of facility-based delivery. Compared to the poorest group, the odds were 1.48 times higher for the poorer group adjusted odds ratio (aOR) = 1.48 (95% confidence interval (CI) = 1.33–1.66), 2.27 times higher for the middle group aOR = 2.27 (95% CI = 1.95–2.64), 3.84 times higher for the rich group aOR = 3.84 (95% CI = 3.13–4.69), and 5.96 times higher for the richest group aOR = 5.96 (95% CI = 4.43–8.02). Women who reside in the Upper East region were more likely to utilise maternal health services.

**Conclusions:**

Ghana’s fee exemption policy positively impacts maternal health service utilisation among pregnant women. However, there still exist disparities across geographical regions and wealth indexes.

Over the past few decades, there has been a notable decline in global maternal and child mortality, largely driven by the policies and initiatives introduced under the Millennium Development Goals (MDGs) and the Sustainable Development Goals (SDGs) [1,2]. Sustainable Development Goals target 3.1 aims to reduce maternal mortality to less than 70 maternal deaths per 100 000 live births by 2030. As of 2020, the global maternal mortality ratio (MMR) was estimated at 233 maternal deaths per 100 000 live births, with sub-Saharan Africa accounting for approximately 67% of these deaths worldwide [2,3]. Maternal health service utilisation has been reported to have a positive impact on maternal and child health outcomes, and the huge burden of maternal deaths, especially in low- and middle-income countries (LMICs), is linked with unequal access to maternal health care services [4]. Available evidence shows that inequalities in income primarily influence the decision of pregnant women to access maternal health services [5,6]. Several studies have reported health insurance policies that exempt user fees as a strategy to improve maternal health service utilisation in many sub-Saharan countries [7–10].

Recent studies done in LMICs reveal that about half of all childbirths do not occur in health facilities [11,12]. In Nigeria, studies report that about 24% of pregnant women accessed their first ANC visit within the first trimester of pregnancy and 54% had the recommended number of ANC visits before delivery [13,14].

Ghana's health insurance system, the National Health Insurance Scheme (NHIS), was established in 2003 and became fully operational by 2005 [15]. Its primary aim is to promote equitable access to health care services for all citizens. The NHIS replaced the previous ‘cash and carry’ system, under which individuals were required to pay the full cost of health care at service delivery facilities [16]. Designed to address most of Ghana's disease burden, the NHIS benefits package includes essential health care services, such as maternity care [17]. Funding for the scheme comes from a combination of premiums, a dedicated health insurance tax, contributions from the Social Security and National Insurance Trust, government allocations, and investment income [17]. Under the NHIS, Ghana implemented a user fee exemption policy for maternal health services in 2008 after the successful piloting of the policy in the Upper East and Upper West Regions [18]. The policy requires pregnant women to register with the NHIS and hold a valid NHIS card at the point of service. The policy provides a full complement of maternal health services, which cover antenatal care visits, free facility-based delivery, and post-natal care services [7,18,19]. Despite the implementation of the maternal health service fee exemption policy in 2008 to remove financial barriers to accessing maternal health services for pregnant women, Ghana reported an MMR of 310 maternal deaths per 100 000 live births in 2022 [7,19]. The reported relatively high MMR seems to suggest a disconnect between the impact of the access improvement policy and the expected maternal health outcomes. This study assessed the impact of the ‘fee exemption policy’ on maternal health service utilisation in Ghana by utilising pooled nationally representative data. We hypothesised that Ghana’s fee exemption policy has no significant positive effect on maternal health service utilisation.

Although some studies have assessed the impact of the fee exemption policy on maternal and child mortality, few studies have explored the impact of the policy on maternal health service utilisation [20–22]. We present the first study that uses robust causal methods to analyse nationally representative data, including the most recent survey, to determine the effect of Ghana’s fee exemption policy on maternal health service utilisation. The findings of this paper also address the gap in the literature of robust causal methods in analysing nationally representative data to determine the impact of Ghana’s fee-exemption policy on maternal health service use.

## METHODS

### Study design and data source

This study used cross-sectional data from three rounds of the Ghana Demographic and Health Survey (GDHS) and one round of the Ghana Maternal and Health Survey (GMHS) conducted by the Ghana Statistical Service in collaboration with the Ministry of Health and supported by USAID. The surveys used in this study are nationally representative surveys performed periodically to collect health indicators distributed across sociodemographic characteristics. The required data for this study were obtained from the official Demographic and Health Survey (DHS) website: http://www.dhsprogram.com by email request. Details of the DHS data sampling method can be found elsewhere [23]. The protocol for DHS surveys is approved by the Ethics Committee of ORC Macro Inc. This study used anonymised secondary data available in the public domain of DHS. The DHS program explicitly seeks the consent of survey respondents. However, the authors obtained approval from DHS for the reuse of the data. We included 19 155 respondents with complete data for exposure, outcome, and independent variables in our study. The breakdown of the sample population is detailed in Figure S1 in the **Online Supplementary Document****.** This study adheres to the STROBE guidelines for reporting observational studies and a STROBE checklist for cross-sectional studies is reported in Appendix S1 in the **Online Supplementary Document****.**

### Study variables

#### Outcome variables

This study examined three indicators of maternal health service use based on the World Health Organization (WHO) and Ghana Health Service (GHS) recommended guidelines on essential maternal care services, including the timing of first antenatal care (ANC) visit, completion of the recommended number of ANC visit and facility-based delivery. The recommendations stipulate that the first ANC visit should be in the first trimester of pregnancy, a minimum of 4 ANC visits during pregnancy, and health facility-based delivery. This study extracted the variables m13 (timing of first antennal check in months), m14 (number of antenatal visits during pregnancy), and m15 (place of delivery) from the original data set of three DHS surveys (2008–2022) and Q409 (timing of first antennal check in months), Q412 (number of antenatal visits during pregnancy), and Q430 (place of delivery) from the 2017 GMHS to construct a binary outcome variable for the timing of first ANC visit, completing the recommended ANC visits during pregnancy and facility-based delivery. Other variables and harmonised coding from the four national surveys used in this study are described in Table S1 in the **Online Supplementary Document****)**.

#### Exposure variables

The fee exemption policy applies to women registered with the NHIS. Therefore, women who were insured with NHIS were classified as the treatment group, and uninsured women were classified as the control group.

#### Independent covariates

This study adjusted for maternal age, marital status, parity, level of education, religion, wealth index, media exposure (Radio and TV), residence type, and region of residence

### Statistical analysis

The analysis was conducted using R version 4.4.1 (R Core Team, Vienna, Austria, 2024). We applied sample weighting to account for the complex design of the DHS data sets. This involved using the primary sampling units and the rural/urban area of residence. Hence, we selected the sample weight variables v005 (GDHS 2008–2022) and QWEIGHT (GMHS 2017), divided them by 1 000 000 to adjust for six decimal places, and used the survey package in subsequent analysis. We used descriptive statistics to summarise the baseline characteristics of the study population using the tableone package. We reported means, standard deviations, and *P*-values using the Wilcoxon rank-sum test for continuous variables. For categorical variables, we presented frequencies and percentages and assessed associations using the χ^2^ (X^2^) test. We performed a multivariate logistic regression to determine the effect of health insurance coverage on the likelihood of maternal health utilisation. We adjusted for maternal age, marital status, parity, level of education, religion, wealth index, media exposure (radio and TV), residence type, and region as independent covariates.

To estimate the causal effects of the ‘fee exemption policy’ on three binary outcomes, we employed the inverse probability of treatment weighting (IPTW) approach. Propensity scores were calculated using a logistic regression model in which enrolment in the NHIS (binary treatment variable) was regressed on a set of potential confounders: maternal age, marital status, parity, level of education, religion, wealth index, media exposure (radio and TV), residence type, and region. The dependent variable was NHIS enrolment, coded as 1 for insured and 0 for uninsured. This selection of confounders was informed by theoretical considerations and prior literature linking these factors to both NHIS enrolment and the outcomes of interest [24,25]. The WeightIt package was used to obtain IPTW weights under the Average Treatment Effect (ATE) estimand, weighted by the survey sampling weight. Using the calculated IPTW weights, we defined a survey design object with the svydesign function to account for the complex survey sampling structure of the GDHS and GMHS survey data sets. The design incorporated both the sampling weights and IPTW weights to accurately reflect the weighted population structure and reduce bias in effect estimation. The ATE of being enrolled in NHIS on the outcomes was assessed using a survey-weighted generalised linear model framework by modelling each outcome as a function of NHIS enrolment using the svyglm function. Robust standard errors were applied to account for potential heteroscedasticity and non-normality of residuals, ensuring more accurate significance testing. The results were interpreted in terms of adjusted odds ratios (aOR), reflecting the likelihood of outcomes for the overall population. Results with *P*-values less than 0.05 were deemed statistically significant

#### Propensity score diagnostics and balance assessment

**Figure 1:**
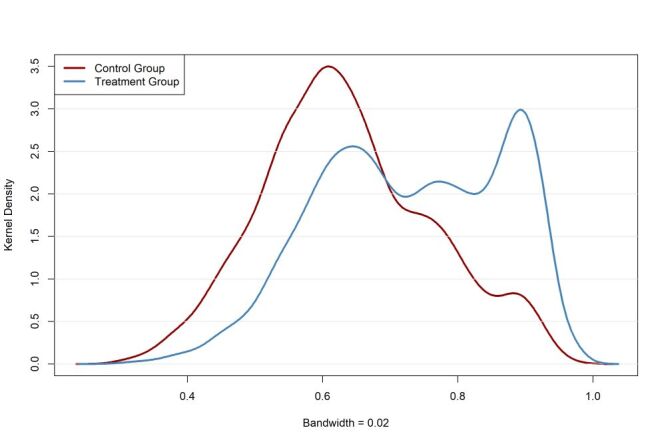
Kernel plot of propensity score by group.

We evaluated the distribution of propensity scores for each group to confirm overlap and verify the weight assignment across the treatment and control groups. A kernel density estimation was applied to plot (**Figure 1**) the distribution of propensity scores using a bandwidth of 0.02 to check for a diagnosis of propensity score. We summarised the covariates balance using the bal.tab function from the cobalt package. Covariate balance was evaluated using standardised mean differences (SMDs) before and after weighting, with an SMD threshold of <0.1 considered indicative of adequate balance. We also utilised a love plot (Figure S2 in the **Online Supplementary Document****)** to visualise the individual SMDs for each covariate and a box plot (Figure S3 in the **Online Supplementary Document**) to view the summary of covariate balance before and after weighting.

## RESULTS

### Descriptive statistics

**Table 1** shows the baseline characteristics of the survey-weighted study population. The mean maternal age was approximately 30.7 ± 7.3 years for the control group of uninsured women and 30.1 ± 6.8 for the treatment group comprising of insured women. The mean parity of the women surveyed in this study was 3.3 ± 2.1 for uninsured women and 3.0 ± 1.9 for insured. The majority of respondents were in unions, either married or cohabiting, and most of the study population had attained secondary education as their highest level of education. Among the unweighted study population, 70.2% were insured with the NHIS and formed the treatment cohort, while 29.8% comprised the control group. The data are presented in Table S2 in the **Online Supplementary Document**.

**Table 1:** Weight-adjusted baseline characteristics of the sample population

Variables	Control, n (%)	Treatment, n (%)	*P*-value
**Survey year**			<0.001
2008	77.2 (1.4)	706.4 (5.8)	
2014	338.8 (6.0)	2376.7 (19.5)	
2017	4176.1 (73.8)	5679.9 (46.7)	
2022	1066.9 (18.9)	3412.1 (28.0)	
**Age, x̄ ± SD**	30.7 ± 7.26	30.1 ± 6.82	<0.001
**Parity, x̄ ± SD**	3.3 ± 2.07	3.0 ± 1.92	<0.001
**Marital status**			<0.001
Single	997.6 (17.6)	1688.0 (13.9)	
Married	2884.5 (51.0)	7621.6 (62.6)	
Cohabitation	1776.8 (31.4)	2865.5 (23.5)	
**Education**			<0.001
No education	1319.0 (23.3)	2697.8 (22.2)	
Primary	1132.5 (20.0)	1942.7 (16.0)	
Secondary	3003.9 (53.1)	6476.1 (53.2)	
Higher	203.6 (3.6)	1058.6 (8.7)	
**Residence**			0.705
Urban	2770.9 (49.0)	6008.2 (49.3)	
Rural	2888.1 (51.0)	6167.0 (50.7)	
**Religion**			
No religion	147.6 (2.6)	216.7 (1.8)	
Christianity	4388.3 (77.5)	9151.5 (75.2)	
Islam	967.5 (17.1)	2579.6 (21.2)	
Traditional	155.6 (2.7)	227.4 (1.9)	
**Wealth index**			<0.001
Poorest	1223.7 (21.6)	2346.7 (19.3)	
Poorer	1274.8 (22.5)	2378.7 (19.5)	
Middle	1152.5 (20.4)	2404.7 (19.8)	
Rich	1087.0 (19.2)	2569.5 (21.1)	
Richest	921.1 (16.3)	2475.7 (20.3)	
**Media exposure (radio)**			0.030
Not at all	1497.6 (26.5)	2947.2 (24.2)	
Less than once a week	1394.6 (24.6)	3141.5 (25.8)	
Every week	2766.8 (48.9)	6086.5 (50.0)	
**Media exposure (TV)**			0.527
Not at all	1462.2 (25.8)	3153.4 (25.9)	
Less than once a week	948.5 (16.8)	2137.3 (17.6)	
Every week	3248.2 (57.4)	6884.5 (56.5)	
**Region**			<0.001
Western*	660.5 (11.7)	1345.5 (11.1)	
Central	570.4 (10.1)	1030.4 (8.5)	
Greater Accra	962.5 (17.0)	1557.0 (12.8)	
Volta†	388.8 (6.9)	914.2 (7.5)	
Eastern	410.2 (7.2)	1268.1 (10.4)	
Ashanti	1152.5 (20.4)	2094.9 (17.2)	
Brong Ahafo‡	547.5 (9.7)	1441.7 (11.8)	
Northern§	584.7 (10.3)	1509.9 (12.4)	
Upper East	222.3 (3.9)	614.4 (5.0)	
Upper West	159.6 (2.8)	399.0 (3.3)	

SD – standard deviation, x̄ – mean

*Western, Western-North.

†Volta, Oti.

‡Bono, Bono-East, Ahafo.

§Northern, Savanna, North-East.

### Association of health insurance enrolment and maternal health service utilisation

Pregnant women insured with NHIS, who were beneficiaries of the fee exemption policy were more likely to deliver in a health facility aOR = 1.21 (95% CI = 1.11–1.33, *P* < 0.05). Women who were in unions, either married aOR = 1.48 (95% CI = 1.33–1.64, *P* < 0.05) or cohabiting aOR = 1.25 (95% CI = 1.11–1.40, *P* < 0.05) were more likely to initiate ANC visits within the first trimester. Education level was associated with an increasing likelihood of facility-based delivery (**Table 2**). Primary school education aOR = 1.27 (95% CI = 1.13–1.43, *P* < 0.05), secondary school education aOR = 1.82 (95% CI = 1.62–2.04, *P* < 0.05), and higher education aOR = 4.94 (95% CI = 2.94–8.30, *P* < 0.05) all showed significant association with facility-based delivery.

**Table 2:** Multivariate logistic regression between insurance enrolment and maternal health service utilisation

Variables	Timing of first ANC visit			Completing recommended ANC visits			Facility-based delivery		
	**aOR**	95% CI*	***P*-value**	**aOR**	95% CI*	***P*-value**	**aOR**	95% CI*	***P*-value**
**NHIS**									
Control	Ref			Ref			Ref		
Treatment	1.03	(0.96–1.11)	0.349	1.10	(0.98–1.24)	0.118	1.21	(1.11–1.33)	<0.001
**Age**	1.02	(1.01–1.03)	<0.001	1.07	(1.05–1.08)	<0.001	1.03	(1.02–1.04)	<0.001
**Parity**	0.88	(0.86–0.90)	<0.001	0.81	(0.78–0.85)	<0.001	0.85	(0.82–0.87)	<0.001
**Marital status**									
Single	Ref			Ref			Ref		
Married	1.48	(1.33–1.64)	<0.001	1.89	(1.61–2.23)	<0.001	1.07	(0.93–1.24)	0.321
Cohabitation	1.25	(1.11–1.40)	<0.001	1.33	(1.12–1.58)	0.001	0.86	(0.74–0.99)	0.049
**Education**									
No education	Ref			Ref			Ref		
Primary	0.97	(0.88–1.07)	0.497	1.00	(0.86–1.17)	0.989	1.27	(1.13–1.43)	<0.001
Secondary	0.92	(0.84–1.00)	0.058	1.22	(1.04–1.42)	0.014	1.82	(1.62–2.04)	<0.001
Higher	1.21	(1.03–1.40)	0.017	2.05	(1.20–3.49)	0.009	4.94	(2.94–8.30)	<0.001
**Residence**									
Urban	Ref			Ref			Ref		
Rural	1.17	(1.08–1.27)	<0.001	1.00	(0.87–1.16)	0.978	0.53	(0.48–0.59)	<0.001
**Religion**									
No religion	Ref			Ref			Ref		
Christianity	1.18	(0.94–1.48)	0.151	1.12	(0.81–1.53)	0.501	1.43	(1.13–1.79)	0.003
Islam	1.13	(0.89–1.42)	0.316	1.03	(0.74–1.42)	0.880	1.35	(1.06–1.71)	0.014
Traditional	0.69	(0.50–0.94)	0.018	0.68	(0.46–0.99)	0.045	0.69	(0.52–0.93)	0.016
**Wealth index**									
Poorest	Ref			Ref			Ref		
Poorer	1.12	(1.01–1.23)	0.031	1.39	(1.20–1.62)	<0.001	1.48	(1.33–1.66)	<0.001
Middle	1.21	(1.07–1.36)	0.002	1.49	(1.23–1.82)	<0.001	2.27	(1.95–2.64)	<0.001
Rich	1.39	(1.21–1.59)	<0.001	2.67	(2.05–3.48)	<0.001	3.84	(3.13–4.69)	<0.001
Richest	1.78	(1.52–2.09)	<0.001	4.14	(2.84–6.03)	<0.001	5.96	(4.43–8.02)	<0.001
**Media exposure (radio)**									
Not at all	Ref			Ref			Ref		
Less than once a week	0.98	(0.90–1.07)	0.709	1.16	(1.00–1.34)	0.056	1.06	(0.94–1.19)	0.316
Every week	1.04	(0.96–1.12)	0.376	1.22	(1.07–1.39)	0.003	1.07	(0.97–1.18)	0.207
**Media exposure (TV)**									
Not at all	Ref			Ref			Ref		
Less than once a week	0.96	(0.87–1.07)	0.482	1.16	(0.98–1.37)	0.078	0.99	(0.87–1.12)	0.868
Every week	1.04	(0.95–1.14)	0.353	1.25	(1.08–1.44)	0.003	1.08	(0.97–1.21)	0.151
**Region**									
Western†	Ref			Ref			Ref		
Central	1.12	(0.95–1.31)	0.173	1.01	(0.73–1.39)	0.968	0.74	(0.59–0.92)	0.007
Greater Accra	0.76	(0.65–0.89)	0.001	0.71	(0.50–1.01)	0.054	1.09	(0.82–1.46)	0.533
Volta‡	1.29	(1.11–1.50)	0.001	0.65	(0.50–0.85)	0.002	0.71	(0.58–0.86)	0.001
Eastern	0.87	(0.75–1.01)	0.074	0.55	(0.42–0.72)	<0.001	0.85	(0.69–1.04)	0.111
Ashanti	1.00	(0.86–1.15)	0.949	0.94	(0.71–1.25)	0.666	1.16	(0.94–1.43)	0.163
Brong Ahafo§	0.90	(0.79–1.03)	0.129	0.93	(0.72–1.19)	0.552	1.34	(1.11–1.61)	0.002
Northern¶	0.78	(0.68–0.90)	0.001	0.82	(0.64–1.05)	0.118	0.77	(0.64–0.91)	0.003
Upper East	1.19	(1.03–1.38)	0.018	1.99	(1.46–2.70)	<0.001	5.08	(4.04–6.38)	<0.001
Upper West	1.23	(1.06–1.42)	0.006	1.10	(0.84–1.45)	0.493	2.20	(1.79–2.68)	<0.001

aOR – adjusted odds ratio, CI – confidence interval, NHIS – National Health Insurance Scheme, TV – Television

*Lower-Upper.

†Western, Western-North.

‡Volta, Oti.

§Bono, Bono-East, Ahafo.

¶Northern, Savanna, North-East.

Women in lower wealth indexes were less likely to initiate ANC within the first trimester of pregnancy, complete four or more ANC visits during pregnancy, or deliver in a health facility. Women in the richest wealth index showed the highest odds for the timing of ANC visits aOR = 1.78 (95% CI = 1.07–1.36, *P* < 0.05), completing four or more ANC visits aOR = 4.14 (95% CI = 2.84–6.03, *P* < 0.05) and facility-based delivery aOR = 5.96 (95% CI = 4.43–8.02, *P* < 0.05). Media exposure showed no significant association with the likelihood of maternal health service utilisation. Women residing in the Upper East aOR = 1.19 (95% CI = 1.03–1.38, *P* < 0.05), Upper West aOR = 1.23 (95% CI = 1.06–1.42, *P* < 0.05), and Volta aOR = 1.29 (95% CI = 1.11–1.50, *P* < 0.05) regions were more likely to initiate ANC visit within the first trimester of pregnancy. Also, women in the Upper East region were more likely to complete the recommended number of ANC visits aOR = 1.99 (95% CI = 1.46–2.70, *P* < 0.05) and deliver in a health facility aOR = 5.08 (95% CI = 4.04–6.38, *P* < 0.05).

### Impact of fee exemption policy on maternal health service utilisation

**Table 3** shows the timing of ANC visits aOR = 1.08 (95% CI = 1.00–1.17, *P* < 0.05) and completion of recommended ANC visits aOR = 1.09 (95% CI = 1.01–1.18, *P* < 0.05) increased by 8 and 9%, respectively. Facility-based delivery aOR = 1.21 (95% CI = 1.11–1.32, *P* < 0.05) from the observed exponentiated coefficient increased by 21%.

**Table 3:** Average treatment effect of fee exemption policy on maternal health service utilisation

Variables	aOR	95% CI*	*P*-value
Timing of first ANC Visit			
*Intercept*	0.49	(0.46–0.52)	<0.001
*NHIS*	1.08	(1.00–1.17)	0.043
Completing recommended ANC visits			
*Intercept*	10.57	(9.53–11.72)	<0.001
*NHIS*	1.09	(1.01–1.18)	0.003
Facility-based delivery			
*Intercept*	3.77	(3.50–4.07)	<0.001
*NHIS*	1.21	(1.11–1.32)	<0.001

ANC – antenatal care, aOR – adjusted odds ratio, CI – confidence interval, NHIS – National Health Insurance Scheme

*Lower-Upper.

## DISCUSSION

The findings of our study show that the fee exemption policy implemented under the National health insurance scheme had a positive impact on maternal health service utilisation. Although the association analysis did not identify a statistically significant relationship between NHIS enrolment and the timing of recommended ANC visits, the average treatment effect analysis revealed a statistically significant 8% increase in initiating ANC within the recommended first trimester of pregnancy. Evidence from this study shows that the impact of the fee exemption policy was more pronounced in the facility-based delivery indicator. The difference in the impact between ANC uptake and facility-based delivery could be attributed to the implementation mechanism of the policy. First, pregnant women are required to register with the NHIS and hold a valid NHIS card at the point of service delivery to access the fee-exemption benefits of the policy. The NHIS cards, which are valid for five years, are subject to annual renewals either through payments of premiums or registration [17,26]. It has been reported elsewhere of weak administrative processes within the NHIS, which results in delays in the issuance of NHIS cards by the scheme for new registrations, and this could significantly affect the timing of women seeking their first ANC care and may also affect the completion of the recommended number of ANC visits [27]. Second, the scheme instituted a minimum thirty-day waiting period for newly registered cards to be activated and accepted at health facilities [28]. This added wait period could account for the lower impact of the fee exemption policy on early pregnancy maternal health service indicators, such as the timing of first ANC visit as compared to facility-based delivery. Based on the current findings, the null hypothesis is rejected, indicating that Ghana’s fee exemption policy positively influences maternal health service utilisation.

The findings are consistent with studies from Ghana and other LMICs [29–33] where most studies have demonstrated a significant association between health insurance policy and increased utilisation of maternal health care services. Also, our findings are consistent with other studies in determining that facility-based delivery utilisation benefits more from the fee exemption policy [29].

Despite the positive impact of the fee exemption policy on maternal health service utilisation, our results also demonstrated a strong positive association between wealth index and utilisation of maternal health service. Our findings reveal that the wealth index shows a stepwise increase in odds ratio across all maternal health service indicators analysed and reflects a pronounced socioeconomic gradient. This marked disparity emphasises systemic inequalities, including affordability and accessibility of maternal health services. It may also reflect financial barriers to accessing health and differences in cultural practices, perceptions of health care quality, and geographical proximity to health care facilities in poorer communities. Systemic inequalities such as poor transportation systems, the non-comprehensive nature of the NHIS, and the poor attitude of health care providers that are pervasive among underprivileged communities have been found to underscore disparities in health service utilisation in Ghana [34]. Mistreatment of women in labour, especially women from lower-income communities at health facilities, has been widely reported, and this could account for the perceptions of health care quality [35–37]. Despite all these other factors, similar findings have been reported in studies conducted across other regions of sub-Saharan Africa on the impact of insurance and fee exemption policies on maternal health service utilisation [38,39]. Studies conducted in Kenya and Tanzania revealed the advent and increase in health insurance coverage was associated with increase in maternal health utilisation and health outcomes [40,41].

Similar to findings in other studies, our study shows that women residing in the Upper East Region were more likely to initiate ANC visits in the first trimester of pregnancy, complete the recommended four ANC visits, and deliver in a health facility [29,42]. The contributing factors to this finding could be explained by the initial piloting of the fee exemption policy in the Upper East Region. This may have led to policy localisation and acceptance among women in the region. Also, the Upper East region has benefitted from other maternal health improvement policy interventions such as the Community-based Health Planning Services (CHPS) project, a primary health accessibility improvement program that was aimed to relocate health centres and health posts from subdistricts to convenient community locations that realised rapid scale up in the region [43,44].

The findings of this study offer compelling evidence of the positive impact of Ghana’s fee exemption policy on maternal health service utilisation and the null hypothesis is rejected. The study has implications for policy review in the implementation strategies for improving maternal health service utilisation and inadvertently improving on maternal health outcomes in Ghana. The mechanisms for registration and renewal of NHIS cards could be reviewed to improve accessibility to the fee exemption policy for pregnant women and further enhance maternal health utilisation.

### Strengths and limitations

Our study utilises extensive and representative national data from three rounds of GDHS and one round of GMHS, enhancing the population-based generalisation of findings. By applying the inverse probability of treatment weighting technique, our analysis retains the entire sample data and hence preserves the statistical power and provides robust estimates. Despite these strengths, our study has several limitations. First, in this study, data on health care utilisation was self-reported, even though the GDHS and GMHS used well-validated and standardised questionnaires in their surveys, there could still exist recall bias. We explicitly acknowledge this limitation in our study and suggest that future research could use more objective measures of health care utilisation, such as electronic health records, to further validate our findings. Second, in this study, respondents with incomplete data were excluded from the analysis, which could introduce bias if the missingness is not random. However, to attenuate this limitation, we utilised pooled data, which increased the sample size and enhanced the robustness of our analysis.

## CONCLUSIONS

This is the first study assessing the impact of Ghana’s fee exemption policy on maternal health service utilisation by employing a robust analysis of the most recent nationally representative data. By employing IPTW, we enhance the validity of our causal inference, ensuring that our findings more accurately reflect the policy's impact. While IPTW provides robust support for causal inference, the findings are subject to the inherent limitations of the observational design of the study. The findings of this study suggest that government initiatives aimed at providing fair access to maternal health services through the current fee exemption policy under the NHIS positively impacted maternal health service utilisation. However, significant socioeconomic disparities exist in access to maternal health services, and enhanced efforts may be needed to bridge the gap in accessibility specifically for women within the lower wealth index. A review of the policy to exempt fees on NHIS registration and NHIS card renewal for low-income groups could further enhance access to service utilisation and reduce the disparity based on economic status.

## Additional material


Online Supplementary Document

## References

[1] Assefa Y Van Damme W Williams OD Hill PS Successes and challenges of the millennium development goals in Ethiopia: lessons for the sustainable development goals. BMJ Glob Health. 2017;2:e000318. 10.1136/bmjgh-2017-000318 29081999 PMC5656143

[2] World Health Organization. Trends in maternal mortality 2000 to 2020: estimates by WHO, UNICEF, UNFPA, World Bank Group and UNDESA/Population Division. Geneva, Switzerland: World Health Organization; 2023. Available: https://www.who.int/publications/i/item/9789240068759. Accessed: 14 September 2024.

[3] Onambele L Guillen-Aguinaga S Guillen-Aguinaga L Ortega-Leon W Montejo R Alas-Brun R Trends, Projections, and Regional Disparities of Maternal Mortality in Africa (1990–2030): An ARIMA Forecasting Approach. Epidemiologia (Basel). 2023;4:322–51. 10.3390/epidemiologia4030032 37754279 PMC10528291

[4] World Health Organization. Inequality monitoring in sexual, reproductive, maternal, newborn, child and adolescent health: a step-by-step manual. Geneva, Switzerland: World Health Organization; 2022. Available: https://www.who.int/publications/i/item/9789240042438. Accessed: 14 September 2024.

[5] Mackenbach JP The persistence of health inequalities in modern welfare states: the explanation of a paradox. Soc Sci Med. 2012;75:761–9. 10.1016/j.socscimed.2012.02.031 22475407

[6] O’Campo P Urquia M Aligning method with theory: a comparison of two approaches to modeling the social determinants of health. Matern Child Health J. 2012;16:1870–8. 10.1007/s10995-011-0935-1 22183165 PMC3488609

[7] The World Bank Group SGD Fund. World Bank Group Partnership Fund for the Sustainable development goals. Annual Report 2019. World Bank Group: Washington, DC, USA; 2019. Available: https://documents1.worldbank.org/curated/en/106391567056944729/pdf/World-Bank-Group-Partnership-Fund-for-the-Sustainable-Development-Goals-Annual-Report-2019.pdf. Accessed: 14 September 2024.

[8] Manthalu G Yi D Farrar S Nkhoma D The effect of user fee exemption on the utilization of maternal health care at mission health facilities in Malawi. Health Policy Plan. 2016;31:1184–92. 10.1093/heapol/czw050 27175033 PMC5035778

[9] Marye DM Debalkie Atnafu D Belayneh M Takele Alemu A User Fee Exemption Policy Significantly Improved Adherence to Maternal Health Service Utilization in Bahir Dar City, Northwest Ethiopia: A Comparative Cross-Sectional Study. Clinicoecon Outcomes Res. 2023;15:775–85. 10.2147/CEOR.S431488 38106643 PMC10722901

[10] Offosse MJ Avoka C Yameogo P Manli AR Goumbri A Eboreime E Effectiveness of the Gratuité user fee exemption policy on utilization and outcomes of maternal, newborn and child health services in conflict-affected districts of Burkina Faso from 2013 to 2018: a pre-post analysis. Confl Health. 2023;17:33. 10.1186/s13031-023-00530-z 37415179 PMC10324224

[11] Moyer CA Dako-Gyeke P Adanu RM Facility-based delivery and maternal and early neonatal mortality in sub-Saharan Africa: a regional review of the literature. Afr J Reprod Health. 2013;17:30–43. 24069765

[12] Adde KS Dickson KS Amu H Prevalence and determinants of the place of delivery among reproductive age women in sub–Saharan Africa. PLoS One. 2020;15:e0244875. 10.1371/journal.pone.0244875 33382825 PMC7774912

[13] Fagbamigbe AF Olaseinde O Fagbamigbe OS Timing of first antenatal care contact, its associated factors and state-level analysis in Nigeria: a cross-sectional assessment of compliance with the WHO guidelines. BMJ Open. 2021;11:e047835. 10.1136/bmjopen-2020-047835 34588242 PMC8479944

[14] Fagbamigbe AF Olaseinde O Setlhare V Sub-national analysis and determinants of numbers of antenatal care contacts in Nigeria: assessing the compliance with the WHO recommended standard guidelines. BMC Pregnancy Childbirth. 2021;21:402. 10.1186/s12884-021-03837-y 34034680 PMC8152343

[15] Blanchet NJ Fink G Osei-Akoto I The effect of Ghana’s National Health Insurance Scheme on health care utilisation. Ghana Med J. 2012;46:76–84. 22942455 PMC3426378

[16] Wireko I Béland D Kpessa-Whyte M Self-undermining policy feedback and the creation of National Health Insurance in Ghana. Health Policy Plan. 2020;35:1150–8. 10.1093/heapol/czaa080 32989440

[17] Wang W, Temsah G, Mallick L. Health insurance coverage and its impact on maternal health care utilization in low-and middle-income countries. Rockville, MD, USA: ICF International; 2014. Available: https://dhsprogram.com/publications/publication-as45-analytical-studies.cfm. Accessed: 14 September 2024.

[18] Anafi P Mprah WK Jackson AM Jacobson JJ Torres CM Crow BM Implementation of fee-free maternal health-care policy in Ghana: perspectives of users of antenatal and delivery care services from public health-care facilities in Accra. Int Q Community Health Educ. 2018;38:259-67. 10.1177/0272684X18763378 29523057

[19] Ghana UNFPA. 2022 Annual Report. Pushing Forward: To safeguard rights and choices. Ghana: United Nations Population Fund; 2022. Available: https://ghana.unfpa.org/en/publications/2022-annual-report-pushing-forward-safeguard-rights-and-choices-0. Accessed: 14 September 2024.

[20] Witter S Garshong B Ridde V An exploratory study of the policy process and early implementation of the free NHIS coverage for pregnant women in Ghana. Int J Equity Health. 2013;12:16. 10.1186/1475-9276-12-16 23446355 PMC3599129

[21] Azaare J Akweongo P Aryeteey GC Dwomoh D Evaluating the impact of maternal health care policy on stillbirth and perinatal mortality in Ghana; a mixed method approach using two rounds of Ghana demographic and health survey data sets and qualitative design technique. PLoS One. 2022;17:e0274573. 10.1371/journal.pone.0274573 36174023 PMC9521900

[22] Adu J Mulay S Owusu MF Reducing maternal and child mortality in rural Ghana. Pan Afr Med J. 2021;39:263. 10.11604/pamj.2021.39.263.30593 34707764 PMC8520428

[23] Corsi DJ Neuman M Finlay JE Subramanian S Demographic and health surveys: a profile. Int J Epidemiol. 2012;41:1602-13. 10.1093/ije/dys184 23148108

[24] Andersen R Newman JF Societal and individual determinants of medical care utilization in the United States. Milbank Q. 2005;83:0-0. 10.1111/j.1468-0009.2005.00428.x 4198894

[25] Kabir MR Adopting Andersen’s behavior model to identify factors influencing maternal healthcare service utilization in Bangladesh. PLoS One. 2021;16:e0260502. 10.1371/journal.pone.0260502 34843566 PMC8629289

[26] Kwarteng A Akazili J Welaga P Dalinjong PA Asante KP Sarpong D The state of enrollment on the National Health Insurance Scheme in rural Ghana after eight years of implementation. Int J Equity Health. 2019;19:4. 10.1186/s12939-019-1113-0 31892331 PMC6938612

[27] Fenny AP Kusi A Arhinful DK Asante FA Factors contributing to low uptake and renewal of health insurance: a qualitative study in Ghana. Glob Health Res Policy. 2016;1:18. 10.1186/s41256-016-0018-3 29202066 PMC5693548

[28] Akweongo P Gadeka DD Aryeetey G Sumboh J Aheto JMK Aikins M Does mobile renewal make health insurance more responsive to clients? A case study of the National Health Insurance Scheme in Ghana. BMJ Glob Health. 2023;7:e011440. 10.1136/bmjgh-2022-011440 38148107 PMC10846841

[29] Azaare J Aninanya GA Abdulai K Adane F Bio RB Hushie M Maternal health care utilization following the implementation of the free maternal health care policy in Ghana: analysis of Ghana demographic and health surveys 2008–2014. BMC Health Serv Res. 2024;24:207. 10.1186/s12913-024-10661-5 38360707 PMC10870471

[30] Anindya K Lee JT McPake B Wilopo SA Millett C Carvalho N Impact of Indonesia’s national health insurance scheme on inequality in access to maternal health services: A propensity score matched analysis. J Glob Health. 2020;10:010429. 10.7189/jogh.10.010429 32566167 PMC7298736

[31] Wang W Temsah G Mallick L The impact of health insurance on maternal health care utilization: evidence from Ghana, Indonesia and Rwanda. Health Policy Plan. 2017;32:366-75. 28365754 10.1093/heapol/czw135 PMC5400062

[32] ComfortABPetersonLAHattLEEffect of health insurance on the use and provision of maternal health services and maternal and neonatal health outcomes: a systematic review. J Health Popul Nutr. 2013;31:81-105.24992805

[33] Dadjo J Ahinkorah BO Yaya S Health insurance coverage and antenatal care services utilization in West Africa. BMC Health Serv Res. 2022;22:311. 10.1186/s12913-022-07698-9 35255895 PMC8899447

[34] Agyemang-Duah W Peprah C Peprah P Barriers to formal healthcare utilisation among poor older people under the livelihood empowerment against poverty programme in the Atwima Nwabiagya District of Ghana. BMC Public Health. 2019;19:1185. 10.1186/s12889-019-7437-2 31462254 PMC6714403

[35] Balde MD Nasiri K Mehrtash H Soumah A-M Bohren MA Irinyenikan TA Labour companionship and women’s experiences of mistreatment during childbirth: results from a multi-country community-based survey. BMJ Glob Health. 2020;5:e003564. 10.1136/bmjgh-2020-003564 33234502 PMC7684665

[36] Ganle JK, Krampah E. Mistreatment of women in health facilities by midwives during childbirth in Ghana: prevalence and associated factors. In: Ana Polona Mivšek, editor. Selected topics in midwifery care. London, UK: Intechopen 2019. pp. 66-85.

[37] Adu-Bonsaffoh K Mehrtash H Guure C Maya E Vogel JP Irinyenikan TA Vaginal examinations and mistreatment of women during facility-based childbirth in health facilities: secondary analysis of labour observations in Ghana, Guinea and Nigeria. BMJ Glob Health. 2021;5:e006640. 10.1136/bmjgh-2021-006640 34789483 PMC8733942

[38] Mekonen AM Kebede N Dessie A Mihret S Tsega Y Wealth disparities in maternal health service utilization among women of reproductive age in Ethiopia: findings from the mini-EDHS 2019. BMC Health Serv Res. 2024;24:1034. 10.1186/s12913-024-11515-w 39243098 PMC11378606

[39] Shanto HH Al-Zubayer MA Ahammed B Sarder MA Keramat SA Hashmi R Maternal Healthcare Services Utilisation and Its Associated Risk Factors: A Pooled Study of 37 Low-and Middle-Income Countries. Int J Public Health. 2023;68:1606288. 10.3389/ijph.2023.1606288 37936874 PMC10625904

[40] Mose GN Orayo JA Health insurance deepening and its impact on maternal healthcare demand in private hospitals in Kenya. Journal of Social Science Research. 2020;16:62-76. 10.24297/jssr.v16i.8855

[41] Anasel MG Komba C Kacholi G Genda EL Chamwali L Mwakasangula E The effects of health insurance on maternal healthcare utilization in Tanzania. Glob Soc Welf. 2024;11:343-57. 10.1007/s40609-024-00345-7

[42] Dzomeku VM Duodu PA Okyere J Aduse-Poku L Dey NEY Mensah ABB Prevalence, progress, and social inequalities of home deliveries in Ghana from 2006 to 2018: insights from the multiple indicator cluster surveys. BMC Pregnancy Childbirth. 2021;21:518. 10.1186/s12884-021-03989-x 34289803 PMC8296527

[43] Awoonor-Williams JK Sory EK Nyonator FK Phillips JF Wang C Schmitt ML Lessons learned from scaling up a community-based health program in the Upper East Region of northern Ghana. Glob Health Sci Pract. 2013;1:117-33. 10.9745/GHSP-D-12-00012 25276522 PMC4168550

[44] Sakeah E Bawah AA Asuming PO Debpuur C Welaga P Awine T Impact of community health interventions on maternal and child health indicators in the upper east region of Ghana. BMC Pregnancy Childbirth. 2023;23:298. 10.1186/s12884-023-05577-7 37118693 PMC10141815

