# Supplementary material for: Impact of Ghana’s fee exemption policy on maternal health service utilisation: an inverse probability of treatment weighting analysis of pooled national data
Source: J Glob Health. 2025 Feb 21;15:04058. doi: 10.7189/jogh.15.04058 (PMC11843519; doi:10.7189/jogh.15.04058)
Supplement: Online Supplementary Document [file jogh-15-04058-s001.pdf]

Figure S1: Sample flow chart of study population

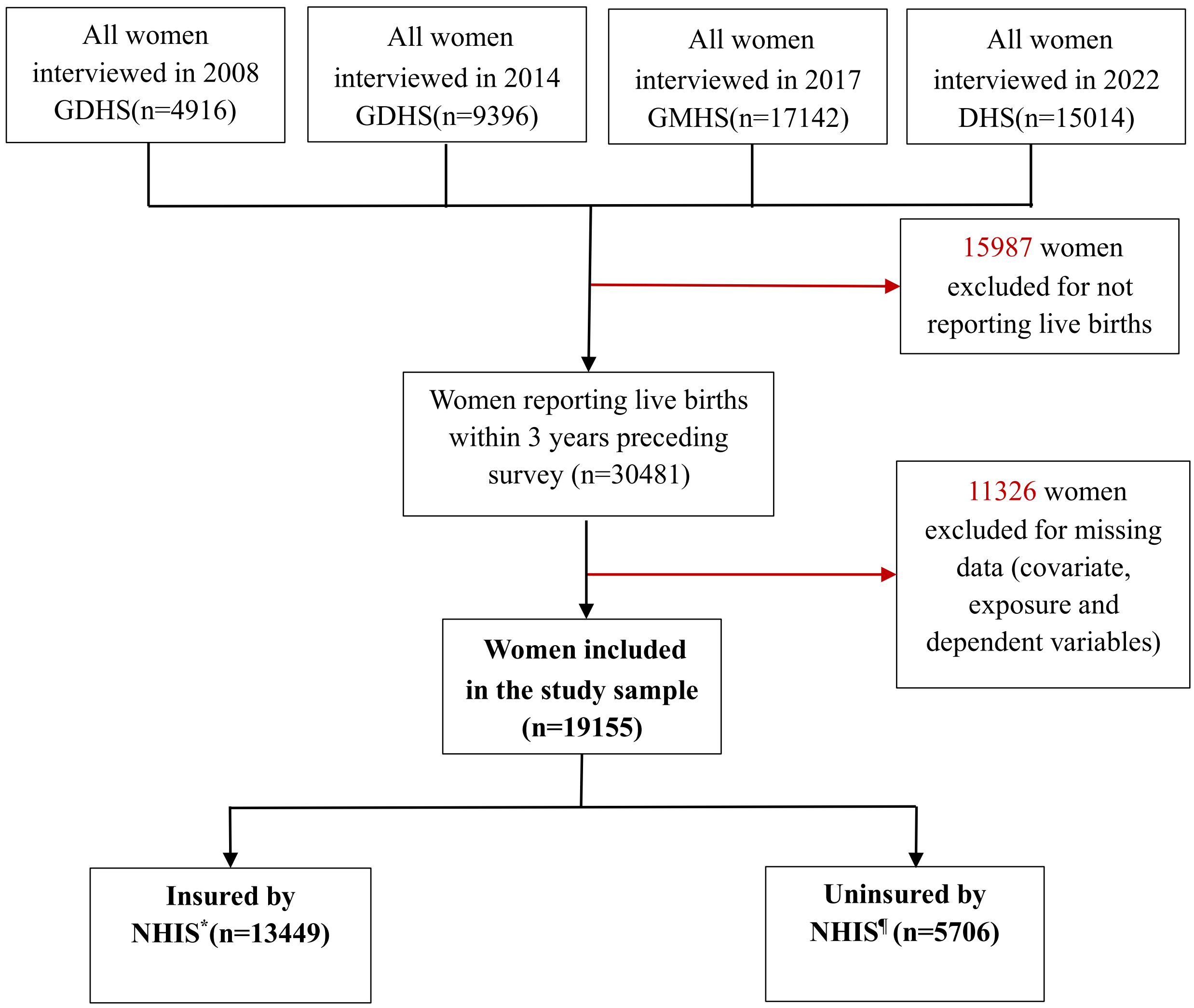

* Treatment group covered by fee exemption policy; ¶ Control group not covered by fee exemption policy

Table S1 List of Variables

Variables	Type	Measurement	Original Variable
**Dependent Variables**			
Timing of ANC visit	Binary	0 No<br>1 Yes	*m13*(DHS 2008-2022)<br>*Q409*(GMHS 2017)
Recommended ANC Visits	Binary	0 No<br>1 Yes	*m14*(DHS 2008-2022)<br>*Q412*(GMHS 2017)
Facility-based delivery	Binary	0 No<br>1 Yes	*m15*(DHS 2008-2022)<br>*Q430*(GMHS 2017)
**Exposure Variable**			
NHIS	Binary	0 No<br>1 Yes	S1015D(GDHS 2008)<br>S1015(GDHS 2014)<br>Q904(GMHS 2017)<br>S1119(GDHS 2022)
**Independent Variables**			
Age	Continuous		*V012* (GDHS 2008-2022)<br>*Q106C* (GMHS2017)
Parity	Continuous		*V201* (GDHS 2008-2022)<br>*Q208A* (GMHS 2017)
Marital status	Categorical	0 Single, divorce, widowed<br>1 Married<br>2 Cohabitation	*V501* (GDHS 2008-2022)<br>*Q701* (GMHS 2017)
Education Level	Categorical	0 No education<br>1 Primary<br>2 Secondary<br>3 Tertiary	*V106* (GDHS 2008-2022)<br>*Q108* (GMHS 2017)
Residence	Binary	1 Urban<br>2 Rural	*V025* (GDHS 2008-2022)<br>*QTYPE* (GMHS 2017)
Wealth Index	Categorial	1 Poorest<br>2 Poorer<br>3 Middle<br>4 Richer<br>5 Richest	*V190* (GDHS 2008-2022)<br>*QWFWLTHI* (GMHS 2017)
Religion	Categorical	0 No religion<br>1 Christianity<br>2 Islam<br>3 Traditional	*V130* (GDHS 2008-2022)<br>*Q122* (GMHS 2017)
Media Exposure (Radio)	Categorical	0 Not at all<br>1 Less than once a week<br>2 Every week	*V158* (GDHS 2008-2022)<br>*Q114* (GMHS 2017)
Media Exposure (TV)		0 Not at all<br>1 Less than once a week<br>2 Every week	*V159* (GDHS 2008-2022)<br>*Q115* (GMHS 2017)
Region	Categorical	1 Western[a]<br>2 Central<br>3 Greater Accra<br>4 Volta[b]<br>5 Eastern	*V024* (GDHS 2008-2022)<br>*QREGION* (GMHS 2017)

	6 Ashanti
	7 Brong Ahafo[c]
	8 Northern[d]
	9 Upper East
	10 Upper West

[a](Western, Western-North), [b](Volta, Oti); [c](Bono, Bono-East, Ahafo); [d](Northern, Savanna, North-East)

Table S2 Baseline characteristics of unweighted sample population (N= 19155)

Variable	Control n (%)	Treatment n (%)	SMD
Total	5706(29.79)	13449(70.21)	
**Survey Year**			0.573
2008	76(1.3)	753(5.6)	
2014	351(6.2)	2549(19.0)	
2017	4108(72.0)	6412(47.7)	
2022	1171(20.5)	3735(27.8)	
**Age***	30.74 (7.3)	30.22 (6.91)	0.073
**Parity***	3.42 (2.2)	3.13 (1.99)	0.141
**Marital Status**			0.198
Single	832 (14.6)	1575 (11.7)	
Married	3447 (60.4)	9378 (69.7)	
Cohabitation	1427 (25.0)	2496 (18.6)	
**Education**			0.263
No Education	1955 (34.3)	4097 (30.5)	
Primary	1139 (20.0)	2167 (16.1)	
Secondary	2462 (43.1)	6109 (45.4)	
Higher	150 (2.6)	1076 (8.0)	
**Residence**			0.061
Urban	2378(41.7)	6008(44.7)	
Rural	3328 (58.3)	7441 (55.3)	
**Religion**			0.125
No Religion	151 (2.6)	262 (1.9)	
Christianity	3848 (67.4)	8958 (66.6)	
Islam	1478 (25.9)	3922 (29.2)	
Traditional	229 (4.0)	307 (2.3)	
**Wealth Quintile**			0.170
Poorest	2010 (35.2)	3943 (29.3)	
Poorer	1273 (22.3)	2834 (21.1)	
Middle	989 (17.3)	2396 (17.8)	
Rich	826 (14.5)	2307 (17.2)	
Richest	608 (10.7)	1969 (14.6)	

**Media Exposure (Radio)**			0.084
Not At All	1844 (32.3)	3836 (28.5)	
Less Than Once a Week	1301 (22.8)	3315 (24.6)	
Every Week	2561 (44.9)	6298 (46.8)	
**Media Exposure (Tv)**			0.039
Not At All	2000 (35.1)	4464 (33.2)	
Less Than Once a Week	935 (16.4)	2281 (17.0)	
Every Week	2771 (48.6)	6704 (49.8)	
**Region**			0.199
Western[a]	499 (8.7)	1201 (8.9)	
Central	391 (6.9)	744 (5.5)	
Greater Accra	508 (8.9)	797 (5.9)	
Volta[b]	340 (6.0)	1001 (7.4)	
Eastern	345 (6.0)	1092 (8.1)	
Ashanti	671 (11.8)	1169 (8.7)	
Brong Ahafo[c]	606 (10.6)	1770 (13.2)	
Northern[d]	1160 (20.3)	2872 (21.4)	
Upper East	612 (10.7)	1427 (10.6)	
Upper West	5740.1)	1376(10.2)	

***Mean ±SD; [a](Western, Western-North), [b](Volta, Oti); [c](Bono, Bono-East, Ahafo); [d](Northern, Savanna, North-East)**

Table S3 Covariate Balance Assessment Before and After Adjustment Using Inverse Probability Treatment Weighting (IPTW)

Balance Measures	Type	Unadjusted SMD	Adjusted SMD	Matching Threshold
Propensity Score	Distance	0.7269	-0.0062	Balanced, <0.1
Age	Contin.	-0.0725	0.0047	Balanced, <0.1
No Education	Binary	-0.038	-0.0122	Balanced, <0.1
Primary Education	Binary	-0.0385	0.0058	Balanced, <0.1
Secondary Education	Binary	0.0228	0.0054	Balanced, <0.1
Higher Education	Binary	0.0537	0.0011	Balanced, <0.1
Parity	Contin.	-0.141	0.0083	Balanced, <0.1
No religion	Binary	-0.007	0.0001	Balanced, <0.1
Christianity	Binary	-0.0083	0.0121	Balanced, <0.1
Islam	Binary	0.0326	-0.0114	Balanced, <0.1
Traditional Religion	Binary	-0.0173	-0.0007	Balanced, <0.1
Not at all (Radio)	Binary	-0.0379	-0.0009	Balanced, <0.1
Less than once a Week(Radio)	Binary	0.0185	0.0026	Balanced, <0.1

Every week (Radio)	Binary	0.0195	-0.0017	Balanced, <0.1
Western[a]	Binary	0.0018	0.0029	Balanced, <0.1
Central	Binary	-0.0132	0.0029	Balanced, <0.1
Greater Accra	Binary	-0.0298	-0.0058	Balanced, <0.1
Volta[b]	Binary	0.0148	0.0092	Balanced, <0.1
Eastern	Binary	0.0207	0.0034	Balanced, <0.1
Ashanti	Binary	-0.0307	0.0032	Balanced, <0.1
Brong Ahafo[c]	Binary	0.0254	0.0053	Balanced, <0.1
Northern[d]	Binary	0.0103	-0.0221	Balanced, <0.1
Upper East	Binary	-0.0012	0.0115	Balanced, <0.1
Upper West	Binary	0.0017	-0.0105	Balanced, <0.1
Single	Binary	-0.0287	0.0045	Balanced, <0.1
Married	Binary	0.0932	-0.0064	Balanced, <0.1
Cohabitation	Binary	-0.0645	0.0018	Balanced, <0.1
Not at all (TV)	Binary	-0.0186	-0.0000	Balanced, <0.1
Less than once a Week(TV)	Binary	0.0057	0.0013	Balanced, <0.1
Every week (TV)	Binary	0.0128	-0.0012	Balanced, <0.1
Residence_2	Binary	-0.0300	-0.0005	Balanced, <0.1
Year 2008	Binary	0.0427	-0.0033	Balanced, <0.1
Year 2014	Binary	0.1280	-0.0026	Balanced, <0.1
Year 2017	Binary	-0.2432	0.0064	Balanced, <0.1
Year 2022	Binary	0.0725	-0.0005	Balanced, <0.1
Poorest	Binary	-0.0591	-0.0062	Balanced, <0.1
Poorer	Binary	-0.0124	0.0014	Balanced, <0.1
Middle	Binary	0.0048	0.0043	Balanced, <0.1
Rich	Binary	0.0268	0.0022	Balanced, <0.1
Richest	Binary	0.0399	-0.0016	Balanced, <0.1

Figure S2: Covariate Balance Before and After Weighting: Absolute Standardized Mean

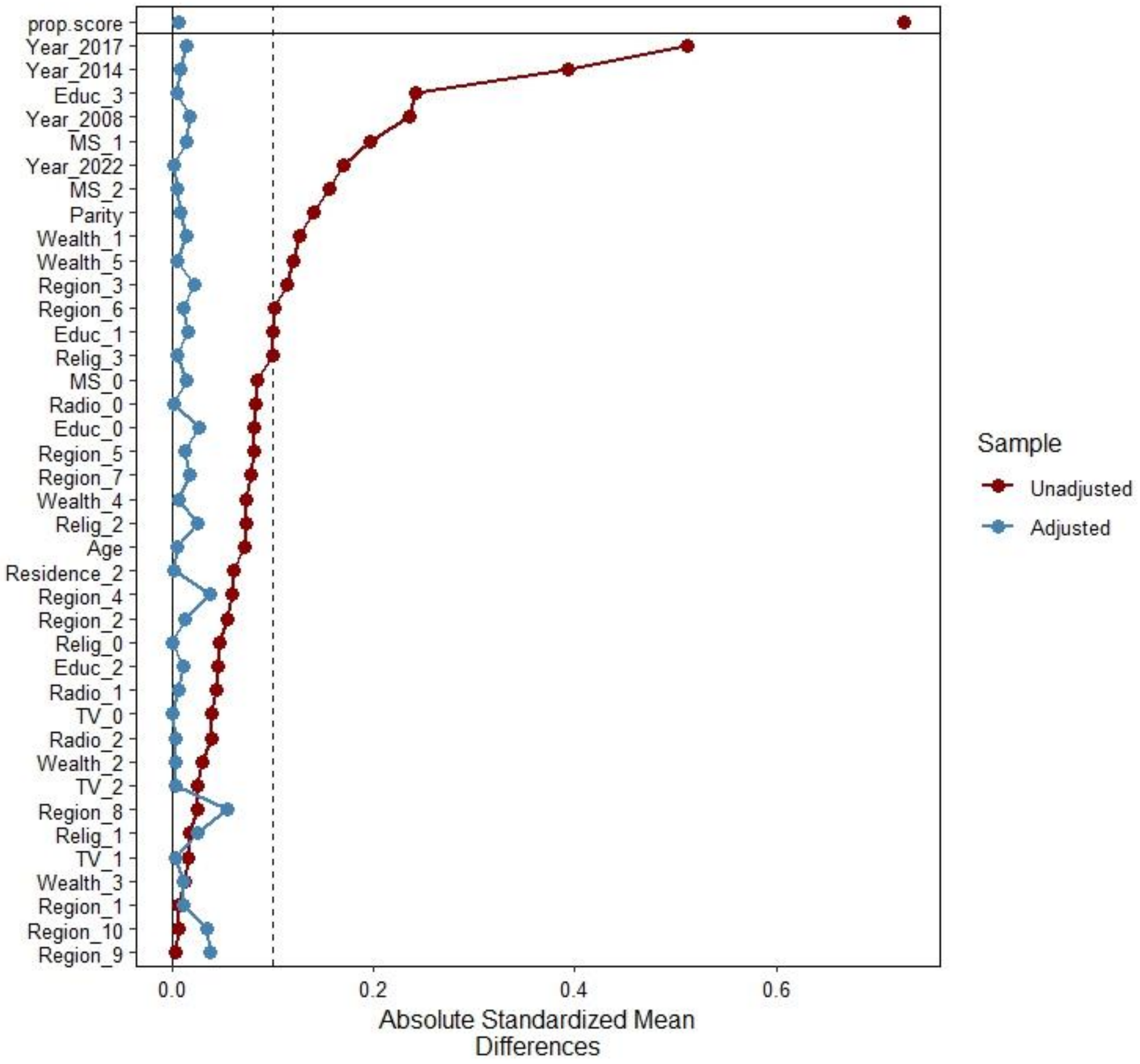

Figure S3: Plot of covariate balance before and after weighting

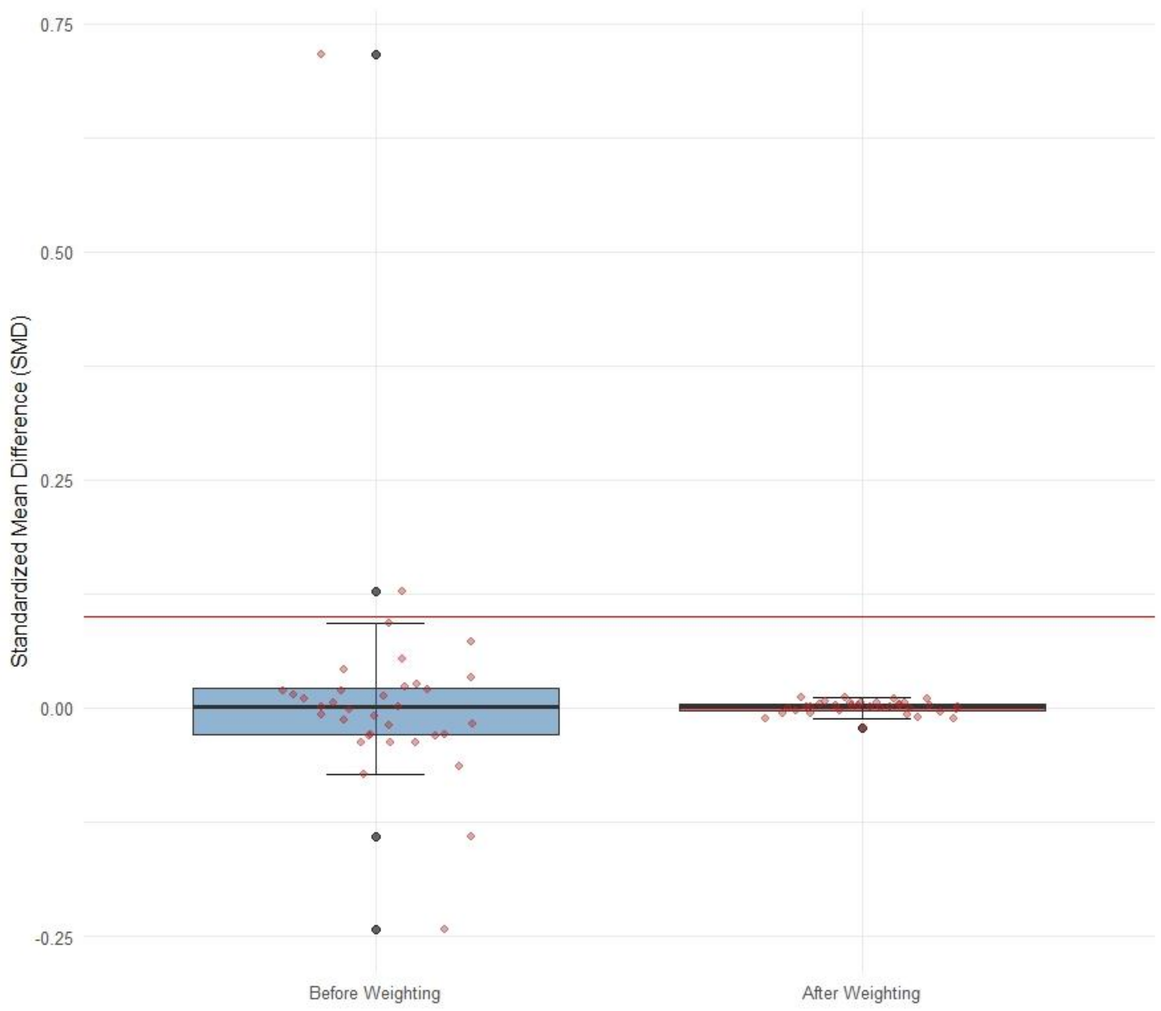

Appendix S1 STROBE Statement

	Item No	Recommendation	Page No
**Title and abstract**	1	(*a*) Indicate the study's design with a commonly used term in the title or the abstract	1
		(*b*) Provide in the abstract an informative and balanced summary of what was done and what was found	2
**Introduction**			
Background/rationale	2	Explain the scientific background and rationale for the investigation being reported	3-4
Objectives	3	State specific objectives, including any prespecified hypotheses	4
**Methods**			
Study design	4	Present key elements of study design early in the paper	4
Setting	5	Describe the setting, locations, and relevant dates, including periods of recruitment, exposure, follow-up, and data collection	4
Participants	6	(*a*) Give the eligibility criteria, and the sources and methods of selection of participants	4
Variables	7	Clearly define all outcomes, exposures, predictors, potential confounders, and effect modifiers. Give diagnostic criteria, if applicable	4-5
Data sources/ measurement	8*	For each variable of interest, give sources of data and details of methods of assessment (measurement). Describe comparability of assessment methods if there is more than one group	4-5
Bias	9	Describe any efforts to address potential sources of bias	5
Study size	10	Explain how the study size was arrived at	4
Quantitative variables	11	Explain how quantitative variables were handled in the analyses. If applicable, describe which groupings were chosen and why	5
Statistical methods	12	(*a*) Describe all statistical methods, including those used to control for confounding	5-6

		(*b*) Describe any methods used to examine subgroups and interactions	6
		(*c*) Explain how missing data were addressed	4
		(*d*) If applicable, describe analytical methods taking account of sampling strategy	5
		(*e*) Describe any sensitivity analyses	6
**Results**			
Participants	13*	(a) Report numbers of individuals at each stage of study—eg numbers potentially eligible, examined for eligibility, confirmed eligible, included in the study, completing follow-up, and analysed	4
		(b) Give reasons for non-participation at each stage	4
		(c) Consider use of a flow diagram	4
Descriptive data	14*	(a) Give characteristics of study participants (eg demographic, clinical, social) and information on exposures and potential confounders	6
		(b) Indicate number of participants with missing data for each variable of interest	6
Outcome data	15*	Report numbers of outcome events or summary measures	6-7
Main results	16	(*a*) Give unadjusted estimates and, if applicable, confounder-adjusted estimates and their precision (eg, 95% confidence interval). Make clear which confounders were adjusted for and why they were included	6-7
		(*b*) Report category boundaries when continuous variables were categorized	7
		(*c*) If relevant, consider translating estimates of relative risk into absolute risk for a meaningful time period	
Other analyses	17	Report other analyses done—eg analyses of subgroups and interactions, and sensitivity analyses	6-7
**Discussion**			
Key results	18	Summarise key results with reference to study objectives	8

Limitations	19	Discuss limitations of the study, taking into account sources of potential bias or imprecision. Discuss both direction and magnitude of any potential bias	8-9
Interpretation	20	Give a cautious overall interpretation of results considering objectives, limitations, multiplicity of analyses, results from similar studies, and other relevant evidence	9
Generalisability	21	Discuss the generalisability (external validity) of the study results	9-10
**Other information**			
Funding	22	Give the source of funding and the role of the funders for the present study and, if applicable, for the original study on which the present article is based	1
